# 蛋白酶体抑制剂纳米靶向给药治疗多发性骨髓瘤研究进展

**DOI:** 10.3760/cma.j.cn121090-20241201-00518

**Published:** 2025-08

**Authors:** 雨昕 林, 多辉 井, 坚青 糜

**Affiliations:** 1 上海交通大学医学院附属瑞金医院血液科，上海血液学研究所，组学与疾病全国重点实验室，国家转化医学研究中心，上海 200025 Department of Hematology, Ruijin Hospital, Shanghai Institute of Hematology, State Key Laboratory of Medical Genomics, National Research Center for Translational Medicine at Shanghai, Shanghai 200025, China; 2 上海血液学研究所，组学与疾病全国重点实验室，国家转化医学研究中心，上海 200025 Shanghai Institute of Hematology, State Key Laboratory of Medical Genomics, National Research Center for Translational Medicine at Shanghai, Shanghai 200025, China; 3 上海市血液病基因编辑与细胞治疗重点实验室（筹），上海 200025 Shanghai Key Laboratory of Gene Editing and Cell-based Immunotherapy for Hematological Diseases, Shanghai 200025, China

## Abstract

近年来，蛋白酶体抑制剂广泛应用于多发性骨髓瘤（MM）的治疗，展现出良好的应用价值，然而，这类药物在临床应用中仍面临诸多挑战，如循环半衰期短、水溶性差、患者耐药及发生严重不良事件。纳米靶向给药凭借其独特优势，有望成为解决上述问题的有效途径之一。本文对国内外蛋白酶体抑制剂的纳米靶向给药机制及其用于MM治疗的研究进展进行梳理，以期为相关领域的研究者提供参考。

## Abstract (en)

In recent years, proteasome inhibitors have been widely applied in the treatment of multiple myeloma（MM）, demonstrating significant therapeutic value. However, these drugs face numerous clinical challenges, such as short circulation half-life, poor water solubility, patient resistance, and severe adverse events. Nanoparticles for targeted drug delivery, with unique advantages, have shown promise as an effective solution to these issues. This paper reviews the mechanisms of nanotargeted drug delivery for proteasome inhibitors and the progress of their use in MM treatment both domestically and internationally, aiming to provide a reference for researchers in related fields.

多发性骨髓瘤（multiple myeloma，MM）是以骨髓中克隆性浆细胞恶性增殖为特征的血液系统恶性肿瘤，我国MM的发病率和死亡率均逐年攀升，当前死亡率达0.63/100 000[1]。美国食品药品监督管理局已批准单克隆抗体、免疫调节剂、蛋白酶体抑制剂等多种药物用于MM的治疗，上述三类药物联合地塞米松的四联疗法适用于大多数初治患者的诱导治疗。

蛋白酶体抑制剂通过诱导错误折叠蛋白积累，引起MM细胞凋亡。自蛋白酶体抑制剂硼替佐米获批用于治疗MM以来，卡非佐米和伊沙佐米也相继获批用于复发/难治性MM的治疗[1]–[2]。和硼替佐米相比，卡非佐米能够不可逆地抑制蛋白酶体，从而增强其疗效，并改善患者的耐受性。然而，这两种药物的临床应用仍然受到循环半衰期较短、水溶性有限、出现耐药性及不良事件严重等因素的限制[3]–[5]。克服上述挑战需要优化制剂中的药物载体，以提高药物的输送效率，延长药效，并减少耐药和其他不良事件的发生[3]。

以纳米颗粒（1个或多个外部尺寸为1～100 nm的颗粒）为载体的靶向给药方式极具潜力[6]。纳米载体的包裹提高了药物的水溶性和稳定性，增强药物穿透细胞膜的能力，并延长了半衰期，减少了药物对正常组织的损害[7]。因此，该药物递送策略被认为可减少不良反应的发生[8]–[9]。研究显示，纳米载体输送硼替佐米和卡非佐米显著提升了MM的治疗效果[10]–[12]。本文综述了目前国内外蛋白酶体抑制剂纳米靶向给药治疗MM研究进展，旨在为相关研究提供参考。

一、基于纳米颗粒的靶向给药机制

1. 基于纳米颗粒靶向给药的总体应用概况：近几十年来，纳米载体用于靶向给药研究引起了广泛关注[6],[13]–[14]。游离药物一般通过血液系统扩散，其在不同组织中的浓度高度依赖于血液流量的分配，但也会受到药物膜转运蛋白、外排泵和代谢酶的影响[15]。与游离药物相比，纳米颗粒通过基团相互作用对药物分子进行包埋[13],[16]，从而使提高了药物的稳定性和生物相容性[17]，而且通过调节纳米材料的物理特性，还能达到靶向某种特定组织和细胞的目的。

2. 常见的靶向给药递送载体：目前，常见的靶向给药递送载体包括脂质体、聚合物胶束、无机纳米粒子等[14]。脂质体是一种球形囊泡，可通过包封药物实现药物负载。聚合物胶束在水溶液中形成具有核壳结构的纳米体系：其中疏水性核心可用于负载疏水性药物，而亲水性壳层则确保体系的水溶性并稳定核心。无机纳米颗粒（如金属纳米颗粒和磁性纳米颗粒）也可通过物理吸附、离子键或共价键结合药物，从而实现药物负载[13],[16],[18]。

3. 靶向机制分类（图1）[19]：在纳米颗粒的靶向机制中，主要存在三种类型：被动靶向、主动靶向和内源性靶向[20]。目前，被动靶向和主动靶向策略已经在蛋白酶体抑制剂治疗MM的研究中得到了广泛应用[11],[21]–[22]。而内源性靶向是基于纳米颗粒结合不同血浆蛋白实现的，在MM治疗领域中与内源性靶向相关的具体应用尚未实施。

**图1:**
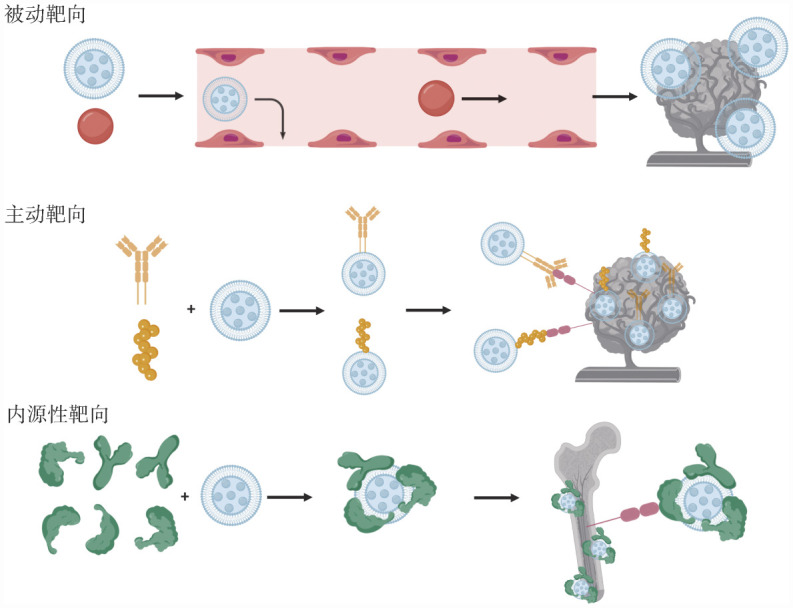
纳米载体靶向给药机制图[19]

被动靶向的实现完全依赖于纳米颗粒的物理特性与肿瘤的病理生理特征的相互作用[23]。在肿瘤治疗的研究中，被动靶向一般是通过高渗透及高保留效应（enhanced permeability and retention，EPR）实现的。由于肿瘤组织快速增生，肿瘤组织内部往往会产生大量不连续、结构异常的血管，这些血管较正常血管具有更高的通透性，使纳米粒子更易到达肿瘤组织内。此外，肿瘤还会引起淋巴系统的功能障碍，并与严重阻碍液体扩散和对流的肿瘤微环境共同作用，使纳米颗粒滞留于肿瘤组织中[24]–[25]。

MM作为血液系统恶性肿瘤，在EPR的既往研究中并未受到充分重视[4]。然而，已有研究表明，在MM等血液肿瘤患者的骨髓中观察到微血管密度增加、血管生成因子及其受体高度表达和炎症细胞浸润，意味着血管生成活跃及毛细血管通透性增加[26]。进一步研究发现，MM患者骨髓中的内皮细胞会形成多孔异常血管[27]。因此，可认为MM患者骨髓具有发生EPR的生物学基础。然而，尽管基于EPR的被动靶向机制可以有效提高药物疗效，减轻药物不良反应，但其精确性和特异性仍有待提高[28]。对于MM和体积较大的其他肿瘤而言，通过EPR在肿瘤中心区域积累的纳米粒子数量有限[4],[29]，导致药物浓度较低，削弱了治疗效果[15]。

国内外的大量研究聚焦于开发更具针对性的靶向给药方式，旨在超越仅依赖肿瘤微环境被动靶向效应[30]，通过特定的表面修饰技术，使纳米颗粒能够特异性识别并结合肿瘤细胞或其微环境，这一过程被称为主动靶向[17]。通过这种方式，纳米载体可以有效地将更高比例的药物直接递送到病变部位，从而改善疗效并降低系统性不良反应。与仅依赖纳米颗粒物理性质的被动靶向相比，纳米颗粒表面修饰物的理化特性在主动靶向中发挥了关键作用[31]。目前，常用的表面修饰物包括配体、抗体、肽类及小分子，这些修饰物已被广泛应用于蛋白酶体抑制剂的纳米靶向递送中，使纳米颗粒能够特异性靶向MM细胞或肿瘤微环境中的相应部位[11]–[12],[32]–[33]。然而，不同修饰物靶向性的差异可能会导致体内纳米颗粒分布不同，从而导致不同的治疗效果[8]。本文将对被动和主动靶向药物的研发及其在MM临床治疗中的应用进行具体阐述。

二、蛋白酶体抑制剂纳米靶向给药的应用

1. 纳米载体被动靶向给药（表1）：

**表1:** 蛋白酶体抑制剂纳米载体被动靶向给药研究

发表年份	作者	药物成分	纳米载体	体外实验	体内实验
2014年	Ashley等[21]	硼替佐米	PEG-脂质体	有效，毒性较低	有效，毒性较低
2023年	Cao等[10]	硼替佐米	聚碳酸酯纳米颗粒	有效	有效，未观察到明显毒性
2015年	Ao等[34]	卡非佐米	PEG-PCL	有效，载体本身无毒性	未进行
2020年	Che等[35]	硼替佐米和5-氮杂-2ʹ-脱氧胞苷	NH2-PEG-PCL	有效	未进行
2014年	Ashley等[22]	卡非佐米和多柔比星	脂质体	有效，毒性较低	有效，毒性较低

**注** PEG：聚乙二醇；PCL：聚己内酯

（1）单药递送：纳米载体可以将一种蛋白酶体抑制剂单独递送至靶向部位。聚乙二醇（PEG）化脂质体具有良好的生物相容性，可用于搭载硼替佐米。制备成的纳米微粒直径约100 nm，满足EPR的最适宜粒径要求（20～200 nm）。Ashley等[21]证明其具有显著的抗MM作用及高载药量、低毒性。此后，硼替佐米的纳米载体给药引起了广泛关注。Cao等[10]用间充质干细胞（临床首选的外泌体来源）将直径约50 nm的聚碳酸酯载药颗粒封装到被称为凋亡囊泡的外泌体中，实现了高效装载，从而进一步降低了肿瘤负荷，减少了MM小鼠模型中的骨和肾破坏。此外，未经囊泡包裹的载体给药仍会引起体重减轻、白细胞减少、出血和肝肾毒性等不良反应，而凋亡囊泡的包被完全消除了硼替佐米的毒性。因此，和游离药物相比，使用纳米载体进行硼替佐米的单药递送能够增强其疗效并减少不良反应。

基于硼替佐米所具有的刺激MM患者成骨细胞分化并抑制其破骨细胞生成的特性，研究者将含硼替佐米的纳米载体加入磷酸钙骨水泥等骨替代材料，以实现硼替佐米的局部缓释，理论上可改善MM导致的溶骨性骨病变。Vehlow等[36]制备了装载硼替佐米的邻苯二酚聚电解质复合物（PEC），将其作为黏合剂涂覆在磷酸钙骨水泥的表面，构建了一种功能化的生物材料，为MM引起的溶骨性骨病变的局部治疗提供了新思路。此外，纳米材料自组装是纳米药物治疗MM领域的一个新热点。自组装指纳米尺度的材料在没有人为干预的情况下自发形成有序结构的过程，基于这一过程而构建的纳米给药系统有助于增强药物对环境的适应性。Ao等[34]使用PEG-聚己内酯（PCL）自组装形成的聚合胶束装载卡非佐米，提高了其代谢稳定性，同时增强了对MM细胞的毒性。和脂质体相比，聚合胶束的粒径更小，更适用于浸润肿瘤组织。

近年来，研究者对卡非佐米自组装纳米给药进行了新探索，如使用自组装三元多肽纳米颗粒作为药物递送载体。和传统的胶束给药系统相比，这一创新策略提高了载药量并降低了载体的免疫原性。然而，此类载体易面临在治疗早期因爆发性药物释放导致药物大量损失的问题[37]–[38]。对此，Jackson等[38]通过在制备过程中添加有机酸成功提高了三元多肽纳米颗粒稳定性，抑制爆发性释放，从而显著延长了药物的半衰期。研究还表明，有机酸的类型和混合比例对于纳米药物的稳定性和药物释放动力学有显著影响。相似地，Agbana等[37]以聚阳离子为稳定剂，通过聚离子络合作用稳定了三元多肽纳米组装体，使其在酸性条件（如肿瘤微环境）下释放卡非佐米。不但有效解决了爆发性药物释放的问题，还显著增强了卡非佐米对肿瘤细胞的靶向作用。这种基于自组装的纳米药物递送策略为提高蛋白酶体抑制剂的治疗效果提供了新思路，并在降低药物不良反应和增强靶向性方面展现出广阔前景。

（2）联合递送：不同抗肿瘤药物能以不同机制作用于肿瘤，或作用于异质性肿瘤内的不同亚群。因此，针对恶性肿瘤的联合用药有助于防止肿瘤耐药现象发生，并能达到优于单药治疗的效果[39]。如硼替佐米、来那度胺和地塞米松（VRd）联合用药方案是新诊断MM患者的首选一线治疗选择[40]。

纳米材料能克服不同药物药代动力学、生物分布和代谢的差异，实现多种药物以最适宜比例递送至肿瘤部位，达到联合用药的最佳疗效[20]，因此，相应药物联合递送的方法有待开发。在MM的纳米靶向治疗中，硼替佐米与其他抗肿瘤药物的联合用药成为重要研究方向之一。Che等[35]以NH_2_-PEG-PCL为载体，通过自组装方式封装硼替佐米和核苷类似物5-氮杂-2ʹ-脱氧胞苷，形成直径约200 nm的纳米颗粒。实验表明，该纳米载体可实现药物缓释，并增强对MM细胞的生长抑制和凋亡诱导。

卡非佐米获批上市后不久，Ashley等[22]首次证明卡非佐米和多柔比星在MM治疗中有协同作用，并在确定游离药物的最佳化学计量比后，将两药精确地按比例掺入脂质体中，形成直径约70 nm的脂质体纳米颗粒。与游离药物的联合疗法相比，这种脂质体给药方法显著提高了MM细胞摄入药物的速率，增强了药物疗效，同时减少了MM小鼠实验中常见的体重减轻等全身性药物不良反应。

2. 纳米载体主动靶向给药：

（1）小分子修饰纳米载体：Swami等[41]设计和合成了结合阿仑膦酸的聚乳酸-羟基乙酸（PLGA）PEG，用以封装硼替佐米，通过阿仑膦酸对骨矿物质的靶向实现了药物的骨归巢及持续的药物释放，同时增强了载体的生物相容性和降解性。MM在细胞膜上表达高水平叶酸受体。尽管叶酸受体在几种正常组织的上皮细胞中也有所表达，但其主要分布在组织的管腔表面，故不易受到静脉内给药的影响。基于此，Nigro等[32]采用叶酸修饰的介孔二氧化硅纳米颗粒很快地将硼替佐米靶向递送到MM细胞，这一方法避免了药物的过早释放和代谢消除，从而显著减少了药物的不良反应。与靶向骨的小分子药物相比，靶向MM细胞的策略进一步优化了纳米颗粒的组织分布，提高了疗效。

（2）选择素修饰纳米载体：选择素家族是一组钙依赖性的细胞黏附分子，包括E-选择素（表达于内皮细胞）、P-选择素（表达于血小板和内皮细胞）及L-选择素[42]。在体内外实验中，通过抑制内皮细胞表面高表达的E-选择素或P-选择素能够破坏MM细胞与骨髓微环境间的相互作用，提升MM对治疗的敏感性[12],[43]。Federico等[12]使用碳化二亚胺化学法将P-选择素糖蛋白配体-1接合到脂质体表面，该载体能够主动靶向内皮细胞，释放其负载的硼替佐米和Rho激酶抑制剂，后者可削弱MM细胞与骨髓微环境的相互作用，从而提高硼替佐米的疗效，并在一定程度上克服骨髓微环境诱导的耐药性。

（3）靶向肽或抗体修饰纳米载体：作为MM免疫治疗的关键靶点，靶向CD138、CD38和BCMA等浆细胞表面受体的纳米载体也已成为研究热点。尽管体外实验中，CD38靶向肽修饰的纳米脂质体在MM细胞摄取率和肿瘤杀伤能力方面不及CD138靶向纳米颗粒，但其在体内表现出更好的生物分布和疗效。可能是因为CD138靶向肽容易与正常淋巴细胞非特异性结合，而CD38靶向肽具有更好的肿瘤特异性[8]。Zhang等[33]使用CD38靶向肽P修饰涂有红细胞膜的磷化三镍纳米颗粒，携载硼替佐米。这种新型载体在体外和体内实验中都被证明能够主动靶向CD38阳性MM细胞，同时显著提升了生物相容性和生物安全性[9]。除靶向肽外，浆细胞表面受体的特异性抗体也已被应用于纳米粒子的主动靶向。de la Puente等[29]将硼替佐米封装于与抗CD38单克隆抗体偶联的交联壳聚糖纳米粒子中。与游离药物或非靶向载体相比，该主动靶向给药系统能有效延缓MM小鼠的肿瘤进展，提高了总生存率并减少了脱发等全身性不良反应，同时未观察到任何器官的组织学毒性。相似地，Chen等[11]使用达雷妥尤单抗修饰装载卡非佐米的胶束，使MM小鼠的中位生存时间较游离药物组延长约2倍。此外，Dutta等[9]构建了与BCMA抗体偶联的PLGA PEG共聚物纳米颗粒。该纳米系统增强了硼替佐米对MM原代细胞的毒性，并减轻了药物对其他细胞的影响，在体内实验中实现了药物在肿瘤部位大量积累，有效减少肿瘤负荷，延长了总生存期。

（4）仿生纳米颗粒同源靶向：硼替佐米的纳米载体主动靶向给药研究已超越了单一受体和配体或抗体之间特异性结合的范畴。其基于细胞膜包覆纳米颗粒形成仿生纳米颗粒，同时继承了其膜来源细胞的特性，细胞膜上保留的抗原谱赋予它们同源靶向等功能。“骨髓归巢”指循环中的MM细胞在其膜表面分子的介导下迁移并返回骨髓的生物学现象。Qu等[4]运用这一机制制备了MM细胞膜包被的硼替佐米纳米载药颗粒，通过同源靶向增强了纳米载体与MM细胞的相互作用，也提高了MM细胞对药物的摄取效率，实现了良好的治疗效果。值得关注的是，该方法可在不影响正常造血细胞的前提下成功靶向并杀伤癌细胞。

三、小结与展望

纳米靶向给药技术在蛋白酶体抑制剂治疗MM的研究中受到广泛关注，为此类药物的递送提供了前景广阔的方法。研究表明，对硼替佐米和卡非佐米进行被动靶向和主动靶向给药均能显著提升两种药物治疗MM的疗效，并减少不良反应。然而，关于卡非佐米纳米靶向给药的研究相对有限，尤其是主动靶向给药策略。蛋白酶体抑制剂治疗MM的纳米给药研究仍存在改进空间，包含靶向机制、适用药物、载体材料等。

1. 新的靶向机制与主动靶向的新靶点：尽管目前关于内源性靶向治疗MM的研究不详，骨髓靶向已成为内源性靶向研究领域的前沿方向。通过增强载体与特定血浆蛋白的结合能力，有望开发出蛋白酶体抑制剂的新型纳米药物[44]。此外，GPRC5D和FcRH5等热门靶点已用于MM的免疫治疗[45]–[46]，但其在主动靶向纳米给药中的应用有待进一步探索。

2. 可使用纳米载体递送的其他蛋白酶体抑制剂：口服药物伊沙佐米的纳米靶向给药相关研究亟待开展。不仅有望优化其临床应用，还可为同样具有口服活性的候选蛋白酶体抑制剂Delanzomib提供参考。另一种新型蛋白酶体抑制剂Marizomib凭借其独特的脑渗透性，已被报道应用于MM脑转移患者的临床治疗[47]，结合能穿越血脑屏障的纳米载体进行递送，可能为中枢神经系统受累的髓外MM治疗带来突破[28],[48]。

3. 可用于联合治疗的新型纳米载体：某些纳米载体能高效递送小干扰RNA，抑制MM进展[43]，该方法在体内实验中已被证明能够延长MM小鼠的生存期。纳米载体在递送蛋白酶体抑制剂时联合递送小干扰RNA将有助于改善患者预后。某些纳米载体具有较高的光热转换效率[5]，在特定波长激光照射下能引发局部温度升高而杀伤肿瘤细胞，使用其递送蛋白酶体抑制剂并联合光热疗法有望成为MM治疗领域的新兴方向。此外，将蛋白酶体抑制剂与来那度胺等免疫调节剂共载于同一纳米载体中，可以通过精准控制药物的递送比例优化MM联合用药的治疗效果。

尽管蛋白酶体抑制剂纳米靶向系统在MM治疗中展现出显著潜力，但多个关键步骤仍需进一步优化，从而达到更高效、更精准的治疗。随着新的药物靶点、作用方式、制备工艺及纳米材料不断涌现，未来有望出现更多纳米靶向疗法。这些创新将极大提升蛋白酶体抑制剂的疗效和安全性，使蛋白酶体抑制剂在治疗MM中发挥更好的作用，为MM患者带来新的希望。
